# Hypertonic saline versus Lactated Ringer’s Solution for neurological recovery in cats with traumatic brain injury

**DOI:** 10.18849/ve.v11i3.743

**Published:** 2026-09-23

**Authors:** Aiden Pang

**Affiliations:** University of Edinburgh United Kingdom

**Keywords:** CATS, FELINE, HEAD TRAUMA, INTRACRANIAL INJURY, NEUROLOGY, TRAUMATIC BRAIN INJURY

## Abstract

**PICO question:**

In cats with traumatic brain injury, is hypertonic saline more effective than Lactated Ringer’s Solution (LRS), when measuring neurological recovery?In dogs diagnosed with canine transmissible venereal tumour (CTVT), does treatment with vincristine combined with ivermectin, compared to treatment with vincristine alone, lead to improved clinical outcomes?

**Clinical bottom line:**

**Category of research:**

Treatment.

**Number and type of study designs reviewed:**

Zero.

**Strength of evidence:**

Zero.

**Outcomes reported:**

Both hypertonic saline and LRS have been successfully used in the management of traumatic brain injuries in cats. There is not enough evidence to recommend one treatment over the other and they should be chosen with consideration of the individual after assessing patient status.

**Conclusion:**

In cats with traumatic brain injury, there is not enough evidence to say that hypertonic saline is more effective than LRS when measuring short-term neurological response with an improved Modified Glasgow Coma Score.

How to apply this evidence in practice

The application of evidence into practice should take into account multiple factors, not limited to: individual clinical expertise, patient’s circumstances and owners’ values, country, location or clinic where you work, the individual case in front of you, the availability of therapies and resources.

Knowledge Summaries are a resource to help reinforce or inform decision making. They do not override the responsibility or judgement of the practitioner to do what is best for the animal in their care.

## The evidence

The literature search found zero evidence that met the inclusion criteria supporting the PICO question asked in this Knowledge Summary.

## Appraisal, application and reflection

Traumatic injury represents 6% of feline cases attending primary veterinary practices in the UK (O’Neill et al., 2023) and is associated with mortality rates ranging from 9–33% (Kolata, 1980; Conroy et al., 2018). Road traffic collisions represent the most frequent incidence of trauma (Hernon et al., 2018), with most cats having multiple injuries. Using the Animal Trauma Triage Score (ATTS), head trauma has been associated with more severe injury and a guarded prognosis (Lee et al., 2022). Prognosis of traumatic brain injury (TBI) with survival to discharge has also been demonstrated using the Modified Glasgow Coma Scale (MGCS) (Cameron et al., 2022), which has been extrapolated from the Glasgow Coma Scale widely used for assessment of TBIs in human patients (Basak et al., 2023).

Intracranial hypertension is a potentially fatal consequence of TBI, associated with a high risk of brain herniation (Dinallo & Waseem, 2023). In dogs, increased intracranial pressure (ICP) can be assessed clinically through the identification of Cushing’s triad: bradycardia, systemic hypertension, and dyspnoea. However, Her et al. (2021) reported that Cushing’s triad is a poor predictor of brain herniation in cats. Instead, decreased levels of consciousness, as assessed by the MGCS, were consistently associated with increased ICP. The majority of cats in that study were diagnosed with intracranial neoplasia and exhibited suspected compensatory responses to increased ICP, complicating the extrapolation of findings to feline TBI. Given these limitations, other parameters are essential for evaluating the severity of TBI and neurological function in cats. These include assessment of consciousness level, mentation, pupil size and reactivity, ocular reflexes, posture, and skeletal motor responses (Garosi & Adamantos, 2011). Furthermore, systemic parameters such as blood pressure, oxygen saturation (SpO_2_), partial pressure of oxygen (PaO_2_) and carbon dioxide (PaCO_2_), blood glucose, and electrolyte balance are critical for maintaining cerebral perfusion pressure (CPP) and monitoring neurological status (Garosi & Adamantos, 2011; Reineke, 2022).

Hypovolaemia is common in TBI patients due to concurrent trauma, and may lead to circulatory shock, impairing cerebral perfusion, and invalidating early neurological assessments (Sande & West, 2010). As such, initial treatment must restore euvolaemia and normotension before a baseline neurological examination is performed. This timing is essential for reliable MGCS assessments and subsequent monitoring of neurological recovery (Platt et al., 2001). Neurological recovery can be defined in various ways: improvement in MGCS scores during hospitalisation (short-term neurological response); survival to discharge (short-term outcome); and long-term resolution of neurological deficits (long-term neurological recovery). This Knowledge Summary adopts short-term neurological improvement, as measured by changes in MGCS or similar parameters during the acute phase of hospitalisation, as the intended outcome measure. Fluid therapy in TBI patients serves two distinct but overlapping purposes: restoring circulating volume to correct shock and reducing intracranial pressure. Varied fluid resuscitation protocols are described in different species (Tranmer et al., 1989; Wisner et al., 1989; Zhuang et al., 1995), and treatment options include isotonic crystalloids such as Lactated Ringer’s Solution (LRS) (Mathews & Parent, 2006) and hyperosmolar solutions like hypertonic saline (HS) or mannitol (Sande & West, 2010). The evidence base for fluid resuscitation in feline TBI is sparse, with no published studies directly comparing LRS and HS in this species. However, understanding the physiological basis, clinical indications, and limitations of both fluids is essential for informed clinical decision making.

Lactated Ringer’s Solution contains sodium (130 mEq/L), chloride, potassium, calcium, and lactate, which acts as a buffer (Singh et al., 2023). Its osmolarity is close to plasma, hence supporting intravascular volume expansion without drawing water from the interstitial or intracellular compartments. Therefore, LRS is typically recommended for the treatment of circulatory shock in dehydrated patients (Krausz, 1995). LRS contains lower sodium concentrations than plasma, therefore may contribute to dilutional hyponatraemia if used in large volumes. Hyponatraemia can exacerbate cerebral oedema, increasing ICP, which is especially concerning in neurologically unstable patients (Byers, 2017).

Hypertonic saline, usually administered as 3% or 7.5% sodium chloride (NaCl) in humans (Mekonnen et al., 2022), is a hyperosmolar solution designed to create an osmotic gradient that shifts fluid from the intracellular and interstitial compartments into the intravascular space. In the context of TBI, this gradient is generated across the blood–brain barrier and reduces brain volume and ICP, improving CPP without requiring large fluid volumes (Tischer & Firth, 2017; Gharizadeh et al., 2022). This is especially advantageous in patients with limited cardiovascular reserve or polytrauma where fluid overload is a risk. In veterinary medicine, however, there are risks associated with HS administration. One is the potential to induce hypernatraemia, which, if not carefully monitored, may lead to complications such as central pontine myelinolysis (CPM). It is important to emphasise that CPM is rare, but most often associated with the rapid correction of severe or chronic hyponatraemia (Laureno, 1983; Mason et al., 2023). This is unlikely in most acute TBI cases, unless there is a pre-existing electrolyte disorder. As such, sodium levels should always be measured before administration of HS, especially of prolonged or repeated dosing is anticipated. Hypertonic saline is often used alongside or following initial fluid resuscitation once euvolaemia has been restored.

Although LRS and HS are frequently used in clinical practice, there is a lack of controlled, comparative studies evaluating their specific effects on neurological recovery in feline TBI. Evidence from other species offers some indirect insight into fluid choice after traumatic brain injury. Simma et al. (1998) conducted a randomised trial in children with severe TBI and found no significant differences in neurological outcomes between fluid types, although respiratory complications were more common in patients resuscitated with LRS. Similarly, experimental studies in dogs (Pinto et al., 2006) have explored the effects of HS on intracranial pressure, with the author favouring HS for its osmotic effects; however, extrapolation to feline patients remains limited.

The feline-specific evidence is limited to isolated case reports and a single experimental study. Ballocco et al. (2019) reported two feline cases with traumatic brain injury: one cat treated with a combination of LRS and HS achieved full neurological recovery, while a second case receiving only HS for intracranial haemorrhage and brain herniation did not survive. Lee and Grady (2024) described a single case of a cat with polytrauma, including TBI, managed with a combination of LRS, HS, and mannitol, resulting in successful neurological recovery; however, the relative contribution of each treatment modality was not assessed. DeWitt et al. (1996) performed an experimental study in cats with induced TBI and intracranial haemorrhage, comparing HS with a colloid solution. Although HS was not found to be superior, the study did not include LRS as a comparator, and results are therefore not generalisable to clinical decision-making between LRS and HS. Collectively, these studies lack the necessary design and sample size to support evidence-based guidelines, and the frequent occurrence of polytrauma complicates interpretation of outcomes. The heterogeneity in TBI aetiologies and coexisting injuries between these individual feline cases further limits the ability to draw conclusions about the comparative efficacy of LRS and HS in feline TBI management.

Given the lack of high-quality, comparative studies in cats, current recommendations are extrapolated from other species and rely heavily on clinical experience and pathophysiological reasoning. In conclusion, LRS is preferentially described as treatment for circulatory shock in dehydrated patients. Once euvolaemia and normotension is achieved, HS is suggested to reduce ICP. Both treatment options have been successfully described in literature. However, there is currently no direct evidence comparing HS and LRS for neurological recovery in cats with TBI, and therefore no conclusive evidence that one is more effective than the other. Clinical decision making has to be extrapolated from studies in other species but considered on an individual basis to address polytrauma and comorbidities. Neurological recovery can be quantified and monitored using MGCS, with prognostic indicators including blood pressure, SpO_2_, PaO_2_, PaCO_2_, respiratory rate/pattern and heart rate.

## Methodology

**Search Strategy:** 

Databases searched and dates covered:	CAB Abstracts on OVID platform [January 2000 – August 2025]PubMed on NCBI platform [January 1965 – August 2025]
Search strategy:	CAB Abstracts: (cat OR cats OR feline) (“traumatic brain injury” OR TBI OR “brain trauma” OR “brain injury” OR “head trauma” OR “head injury”) (fluid OR hypertonic OR “sodium chloride” OR NaCl OR hartmann OR hartmanns OR “sodium lactate” OR isotonic OR ringer OR ringers) (recovery OR neurolog* OR “intracranial pressure” or “intracranial hypertension” OR intracranial OR coma OR “Glasgow coma score” OR “Glasgow coma scale”) 1 and 2 and 3 and 4 PubMed:(cat OR cats OR feline) AND (“traumatic brain injury” OR TBI OR “brain trauma” OR “brain injury” OR “head trauma” OR “head injury”) AND (fluid OR hypertonic OR “sodium chloride” OR NaCl OR hartmann OR hartmanns OR “sodium lactate” OR isotonic OR ringer OR ringers) AND (recovery OR neurolog* OR “intracranial pressure” OR “intracranial hypertension” OR intracranial OR coma OR “Glasgow coma score” OR “Glasgow coma scale”)
Dates searches performed:	4 August 2025

**Exclusion / Inclusion Criteria:** 

Exclusion:	Articles not relevant to the clinical question, inaccessible, not available in English, or books and review articles.
Inclusion:	Primary research studies of any design, available in full text in English, which reported on cats with traumatic brain injury; in which hypertonic saline was administered and its effect could be compared against LRS, either as a designated control/comparator group or within the same study population.

**Search Outcome:** 

Database	Number of results	Excluded – not relevant to the PICO	Excluded – inaccessible	Excluded – non-English language	Excluded – books, reviews, articles	Total relevant papers
Cab Abstracts	6	6	0	0	0	0
PubMed	67	65	1	0	1	0
Total relevant papers when duplicates removed						0

## ORCiD

Aiden Pang: 0009 0008 8484 9787

## Conflict of Interest

The authors declare no conflicts of interest.

## Disclaimer

Knowledge Summaries are a peer-reviewed article type which aims to answer a clinical question based on the best available current evidence. It does not override the responsibility of the practitioner. Informed decisions should be made by considering such factors as individual clinical expertise and judgement along with patient’s circumstances and owners’ values. Knowledge Summaries are a resource to help inform and any opinions expressed within the Knowledge Summaries are the author's own and do not necessarily reflect the view of the RCVS Knowledge. Authors are responsible for the accuracy of the content. While the Editor and Publisher believe that all content herein are in accord with current recommendations and practice at the time of publication, they accept no legal responsibility for any errors or omissions, and make no warranty, express or implied, with respect to material contained within. For further information please refer to our Terms of Use.

## References

[BIBR-1] Ballocco I., Evangelisti M.A., Deiana R., Cubeddu F., Parpaglia M.L.P., Serra G., Carta G., Manunta M.L. (2019). A pilot study evaluating the effect of mannitol and hypertonic saline solution in the treatment of increased intracranial pressure in 2 cats and 1 dog naturally affected by traumatic brain injury. Journal of Veterinary Emergency and Critical Care.

[BIBR-2] Basak D., Chatterjee S., Attergrim J., Sharma M.R., Soni K.D., Verma S., Gerdin Wärnberg M., Roy N. (2023). Glasgow coma scale compared to other trauma scores in discriminating in-hospital mortality of traumatic brain injury patients admitted to urban Indian hospitals: A multicentre prospective cohort study. Injury.

[BIBR-3] Byers C.G. (2017). Fluid Therapy: Options and Rational Selection. Veterinary Clinics of North America: Small Animal Practice.

[BIBR-4] Cameron S., Weltman J.G., Fletcher D.J. (2021). The prognostic value of admission point-of-care testing and modified Glasgow Coma Scale score in dogs and cats with traumatic brain injuries (2007–2010): 212 cases. Journal of Veterinary Emergency and Critical Care.

[BIBR-5] Conroy M., O’Neill D.O., Boag A., Church D., Brodbelt D. (2018). Epidemiology of road traffic accidents in cats attending emergency-care practices in the UK. Journal of Small Animal Practice.

[BIBR-6] DeWitt D.S., Prough D.S., Deal D.D., Vines S.M., Hoen H. (1996). Hypertonic saline does not improve cerebral oxygen delivery after head injury and mild haemorrhage in cats. Critical Care Medicine.

[BIBR-7] Dinallo S., Waseem M. (2023). Cushing Reflex. https://www.ncbi.nlm.nih.gov/books/NBK549801/.

[BIBR-8] Garosi L., Adamantos S. (2011). Head Trauma in the Cat: 2. Assessment and Management of Traumatic Brain Injury. Journal of Feline Medicine and Surgery.

[BIBR-9] Gharizadeh N., Ghokazadeh M., Naseri A., Dolati S., Tarighat F., Soleimanpour H. (2022). Hypertonic saline for traumatic brain injury: a systematic review and meta-analysis. European Journal of Medical Research.

[BIBR-10] Her J., Merbl Y., Gerken K., Kim M., Hofmeister E., Bacek L.M., Kuo K.W., Yanke A.B. (2022). Relationship between admission vitals and brain herniation in 32 cats: A retrospective study. Journal of Feline Medicine and Surgery.

[BIBR-11] Hernon T., Gurney M., Gibson S. (2018). A retrospective study of feline trauma patients admitted to a referral centre. Journal of Small Animal Practice.

[BIBR-12] Kolata R.J. (1980). Trauma in Dogs and Cats: An Overview. The Veterinary Clinics of North America: Small Animal Practice.

[BIBR-13] Krausz M.M. (1995). Controversies in Shock Research: Hypertonic Resuscitation—Pros and Cons.

[BIBR-14] Laureno R. (1983). Central pontine myelinolysis following rapid correction of hyponatremia. Annals of Neurology.

[BIBR-15] Lee E.L., Grady Bailin H. (2024). Washing machine-induced trauma and coagulopathy in a kitten. Canadian Veterinary Journal = La Revue vétérinaire canadienne.

[BIBR-16] Lee J.A., Huang C.-M., Hall K.E. (2022). Epidemiology of severe trauma in cats: An ACVECC VetCOT registry study. Journal of Veterinary Emergency and Critical Care.

[BIBR-17] Mason A., Malik A., Ginglen J.G. (2023). Hypertonic Fluids. https://www.ncbi.nlm.nih.gov/books/NBK542194/.

[BIBR-18] Mathews R., Parent J., Mathews K.A. (2006). Head trauma. Veterinary Emergency and Critical Care Manual.

[BIBR-19] Mekonnen M., Ong V., Florence T., Mozaffari K., Mahgerefteh N., Rana S., Duong C., Plurad D., Yang I. (2022). Hypertonic Saline Treatment in Traumatic Brain Injury: A Systematic Review. World Neurosurgery.

[BIBR-20] O’Neill D., Gunn-Moore D., Sorrell S., McAuslan H., Church D.B., Pegram C., Brodbelt D.C. (2023). Commonly diagnosed disorders in domestic cats in the UK and their associations with sex and age. Journal of Feline Medicine and Surgery.

[BIBR-21] Pinto F.C.G., Capone-Neto A., Prist R., Silva M.R., Poli-de-Figueiredo L.F. (2006). Volume Replacement with Lactated Ringer’s or 3% Hypertonic Saline Solution during Combined Experimental Hemorrhagic Shock and Traumatic Brain Injury. Journal of Trauma: Injury, Infection & Critical Care.

[BIBR-22] Platt S.R., Radaelli S.T., McDonnell J.J. (2001). The Prognostic Value of the Modified Glasgow Coma Scale in Head Trauma in Dogs. Journal of Veterinary Internal Medicine.

[BIBR-23] Reineke E., Drobatz K.J., Reineke E., Costello M.F., Culp W.T.N. (2022). Shock. Feline Emergency and Critical Care Medicine.

[BIBR-24] Sande A., West C. (2010). Traumatic brain injury: a review of pathophysiology and management. Journal of Veterinary Emergency and Critical Care.

[BIBR-25] Simma B., Burger R., Falk M., Sacher P., Fanconi S. (1998). A prospective, randomized, and controlled study of fluid management in children with severe head injury: Lactated Ringer’s solution versus hypertonic saline. Critical Care Medicine.

[BIBR-26] Rout P., Patel P., Davis D. (2025). Crystalloid Solutions in Intravenous Fluid Therapy. https://www.ncbi.nlm.nih.gov/books/NBK500033/.

[BIBR-27] Tischer A., Firth A. (2017). Is the Use of Hypertonic Saline Effective in Reducing Intracranial Pressure After Traumatic Brain Injury in Dogs?. Veterinary Evidence.

[BIBR-28] Tranmer B.I., Iacobacci R.I., Kindt G.W. (1989). Effects of crystalloid and colloid infusions on intracranial pressure and computerized electroencephalographic data in dogs with vasogenic brain edema. Neurosurgery.

[BIBR-29] Wisner D., Busche F., Sturm J., Gaab M., Meyer H. (1989). Traumatic shock and head injury: effects of fluid resuscitation on the brain. The Journal of Surgical Research.

[BIBR-30] (1995). Colloid infusion after brain injury: effect on intracranial pressure, cerebral blood flow, and oxygen delivery. Critical Care Medicine.

